# Measurement of T1 Mapping in Patients With Cardiac Devices: Off-Resonance Error Extends Beyond Visual Artifact but Can Be Quantified and Corrected

**DOI:** 10.3389/fcvm.2021.631366

**Published:** 2021-01-29

**Authors:** Anish N. Bhuva, Thomas A. Treibel, Andreas Seraphim, Paul Scully, Kristopher D. Knott, João B. Augusto, Camilla Torlasco, Katia Menacho, Clement Lau, Kush Patel, James C. Moon, Peter Kellman, Charlotte H. Manisty

**Affiliations:** ^1^Institute for Cardiovascular Science, University College London, London, United Kingdom; ^2^Department of Cardiovascular Imaging, Barts Heart Centre, Barts Health NHS Trust, London, United Kingdom; ^3^Istituto Auxologico Italiano (IRCCS), Istituto Auxologico Italiano, Milan, Italy; ^4^National Institutes of Health, Bethesda, MD, United States

**Keywords:** T1 mapping, MOLLI, cardiac implantable device, aortic valve replacement, cardiovascular magnetic resonance

## Abstract

**Background:** Measurement of myocardial T1 is increasingly incorporated into standard cardiovascular magnetic resonance (CMR) protocols, however accuracy may be reduced in patients with metallic cardiovascular implants. Measurement is feasible in segments free from visual artifact, but there may still be off-resonance induced error.

**Aim:** To quantify off-resonance induced T1 error in patients with metallic cardiovascular implants, and validate a method for error correction for a conventional MOLLI pulse sequence.

**Methods:** Twenty-four patients with cardiac implantable electronic devices (CIEDs: 46% permanent pacemakers, PPMs; 33% implantable loop recorders, ILRs; and 21% implantable cardioverter-defibrillators, ICDs); and 31 patients with aortic valve replacement (AVR) (45% metallic) were studied. Paired mid-myocardial short-axis MOLLI and single breath-hold off-resonance field maps were acquired at 1.5 T. T1 values were measured by AHA segment, and segments with visual artifact were excluded. T1 correction was applied using a published relationship between off-resonance and T1. The accuracy of the correction was assessed in 10 healthy volunteers by measuring T1 before and after external placement of an ICD generator next to the chest to generate off-resonance.

**Results:** T1 values in healthy volunteers with an ICD were underestimated compared to without (967 ± 52 vs. 997 ± 26 ms respectively, *p* = 0.0001), but were similar after correction (*p* = 0.57, residual difference 2 ± 27 ms). Artifact was visible in 4 ± 12, 42 ± 31, and 53 ± 27% of AHA segments in patients with ILRs, PPMs, and ICDs, respectively. In segments without artifact, T1 was underestimated by 63 ms (interquartile range: 7–143) per patient. The greatest error for patients with ILRs, PPMs and ICDs were 79, 146, and 191 ms, respectively. The presence of an AVR did not generate T1 error.

**Conclusion:** Even when there is no visual artifact, there is error in T1 in patients with CIEDs, but not AVRs. Off-resonance field map acquisition can detect error in measured T1, and a correction can be applied to quantify T1 MOLLI accurately.

## Introduction

Parametric mapping using cardiovascular magnetic resonance (CMR) permits non-invasive quantitative myocardial tissue characterization. T1 quantification, performed by sampling the longitudinal relaxation to steady-state, is a useful tool to evaluate patients undergoing CMR for suspected myocardial pathology (1). In addition, it provides unique insight into patients with infiltrative disease, including cardiac amyloidosis, Anderson-Fabry disease and iron overload. Measurement of T1 can assist with diagnosis and clinical decision-making, but these diseases are all associated with cardiac implantable electronic device (CIED) implantation (2–4). CMR can now be performed in nearly all circumstances in patients with CIEDs at 1.5 T, and anatomical cine imaging is diagnostic in most cases (5, 6). The presence of artifact, however, limits the potential of quantitative assessment in a patient group where the clinical yield is high and structural abnormalities are common (7, 8).

The modified Look-Locker inversion recovery (MOLLI) sequence using a balanced steady-state free precession (SSFP) readout is a widely used, commercially available, T1 mapping method and normally has high precision and reproducibility (9, 10). Off-resonance frequency is not widely appreciated as a cause of T1 error, but the presence of metallic artifact can induce large off-resonance effects, affecting the sampled inversion recovery curve. The error may extend beyond visual artifact, making assessment of segments that seem interpretable challenging, because abnormal values can be potentially confused with regional variation due to pathology (11). This may also be important for patients with smaller devices such as implantable loop recorders (ILRs) where visually apparent artifact may be minimal and is therefore not considered such a problem. The presence of sternal wires and metallic valve prostheses may also introduce error into measured T1 that is below the limits of visual detection (12).

The relationship between off-resonance frequency and changes to measured T1 values has previously been described (11), with myocardial T1 values underestimated by 50 ms with off-resonance of ±100 Hz. Measurement of off-resonance frequency can be estimated from a single breath-hold sequence and represented on a voxel-wise color map, enabling assessment of the accuracy of T1 measurement in patients with metallic implants (13, 14).

We hypothesized that the extent of error in T1 is beyond the visual artifact generated by a metallic cardiovascular implant, and is relative to the size of the implant. In patients with either CIEDs or aortic valve replacements (AVRs), we therefore aimed to quantify the off-resonance frequency induced T1 MOLLI error. By generating CIED metallic artifact in healthy volunteers, we also tested the hypothesis that a T1 correction curve for off-resonance frequency, previously derived by Bloch simulations, would permit accurate T1 MOLLI quantification in the presence of off-resonance artifact.

## Methods

### Validation of T1 Error Correction for Off-Resonance With an Externally-Placed ICD in Healthy Volunteers

Ten healthy volunteers were recruited to assess the impact of off-resonance from the presence of a CIED on T1 values, and to test whether correction for off-resonance enabled accurate T1 measurement. The study was approved by the UK National Research Ethics Service (07/H0715/101); conformed to the principles of the Helsinki Declaration, and all subjects gave written informed consent. Body surface area (BSA) was measured before the CMR study. All CMR scans were performed at 1.5 Tesla (Magnetom Aera, Siemens, Erlangen, Germany). After pilot imaging, a second order shim was performed over a volume encompassing the left ventricle. A mid-ventricular short-axis MOLLI 5s(3s)3s map (Siemens MyoMaps) was then acquired (15, 16). In brief, T1 maps were reconstructed from a sequence using the following typical parameters: single breath-hold; slice thickness 8 mm; echo time 1.09 ms; echo spacing 2.7 ms; flip angle 35°; matrix 256 × 144. Off-resonance was then estimated using a single breath-hold ECG-gated field map as previously described, using the same field of view and slice orientation (13). This sequence uses a multiple-echo GRE approach to provide an online voxel-wise representation of residual off-resonance frequency, as a byproduct of a fat water separated image reconstruction (Supplementary Figure 1).

Following acquisition of the initial “reference” T1 map and field map, an implantable cardioverter-defibrillator (ICD) generator (Unify Assura, St Jude Medical Inc., St Paul, MN, USA) was secured to a weighted phantom and placed adjacent to the left chest wall without performing a repeat isocenter. Positioning of the ICD generator was adjusted to mimic off-resonance frequency similar to that expected in patients with CIEDs. Repeat paired MOLLI and field maps were acquired in the same slice position as the reference imaging.

### Patients With Metallic Cardiovascular Implants

To evaluate the extent of T1 error and the application of an error correction, 24 patients with CIEDs and 31 patients with AVRs who were undergoing CMR tissue characterization were studied. Clinical indications were typically for suspected cardiomyopathy or cardiac sarcoidosis, for assessment of substrate for arrhythmia or viability imaging. Paired MOLLI and field maps were acquired using the same protocol as described for the healthy volunteers above. For patients with AVRs, additional paired maps were acquired in the base and apical short-axis orientations. Post contrast T1 mapping was not routinely acquired in patients and so the accuracy of extra-cellular volume fraction was not studied. The CMR safety protocol for patients with CIEDs has been described elsewhere and is in accordance with international guidance (8, 17). In brief, all patients underwent device interrogation and programming immediately prior to CMR. Patients underwent CMR in Normal Operating Mode (SAR limit <2 W/kg) with continuous ECG and pulse oximetry monitoring. Immediately after scan completion, devices were re-interrogated and programmed back to normal settings.

### Analysis

**Figure 1:**
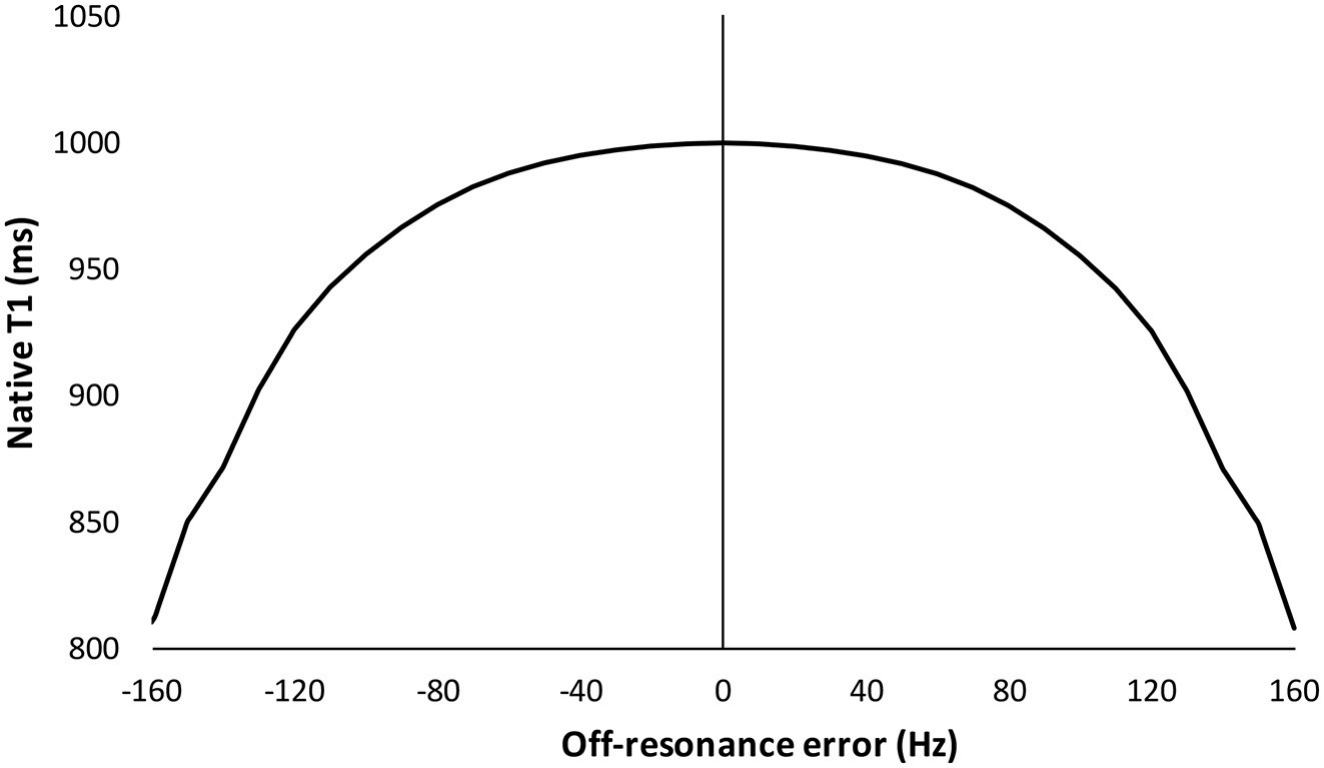
Predicted influence of off-resonance on measured native T1 using Bloch simulation. Simulation is for a standard MOLLI 5(3s)3 sampling scheme with a 35 degree flip angle. Data are estimated for an average expected myocardial T1 of 1,000 ms as per, as per Kellman et al. (11).

Because the presence of an ICD also results in artifact manifest as SSFP hyperintensity bands or signal void, segments with visual artifact were excluded from analysis (18). As previously described, Bloch simulation developed T1 correction curves up to ±160 Hz off-resonance (Figure 1), a range that is expected to be free from banding artifact, and so any segments with greater off-resonance were not corrected (11).

Image analysis was performed using CVI42 (Circle Cardiovascular Imaging Inc., Calgary, Canada). For each T1 map, endocardial and epicardial borders were manually contoured and the superior and inferior RV insertion points were marked to measure T1 in six segments according to the American Heart Association (AHA) classification. A conservative 30% erosion was applied to both borders, avoiding error from endocardial blood-pool partial voluming and increased off-resonance at the epicardial tissue-air interface (19). Because the field map has few anatomical features, the contours were copied and checked on the water separated image reconstruction acquired in same breath-hold as the field map. If necessary, contours were adjusted for small differences in breath-hold or subsequent motion correction depth, and then copied to the field map itself (13). The mean T1 and off-resonance for each AHA segment was then calculated. Both positive and negative off-resonance similarly lower T1 and so the absolute off-resonance frequency values were used to correct T1 using previously described Bloch simulation (Figure 1). The number of segments with significant off-resonance frequency >85 Hz were also analyzed. This cut-off for acceptable accuracy is equivalent to the standard deviation (sd) of the inter-study difference in T1 of 28 ms. This has been previously reported in 51 subjects scanned on two consecutive occasions (20 healthy volunteers, 21 Fabry disease, five cardiac amyloidosis, two aortic stenosis, three cardiomyopathies, age 51 ± 15 years, 20 male) (20).

Whilst measuring segmental artifact is straightforward and its ease facilitates clinical use, an off-line tool was also developed for illustrative purposes using Matlab R2013 (The MathWorks, Inc., Natick, MA, USA). T1 correction based on off-resonance was calculated from field maps and integrated into the automated tool to produce voxel-wise corrected T1 maps (Supplementary Figure 1).

### Statistics

Analysis was performed in Excel (*Microsoft Office 2010, Microsoft, Redmond, WA*) and SPSS version 22 (*IBM Corporation, Armonk, NY, USA*). Data are presented as mean ± sd or median (inter-quartile range, IQR) according to distribution. Categorical variables are presented as absolute numbers or frequencies. A paired Student's *t*-test was used for within group comparisons. Repeated measures and one-way ANOVA was used for multiple measurements from the same and between different groups, respectively. Pearson's correlation coefficient (*r*) was used to assess correlation. All tests were two-tailed and *p* < 0.05 was considered statistically significant.

## Results

### Validation of T1 Error Correction in Healthy Volunteers Using an Externally Placed CIED

**Figure 2:**
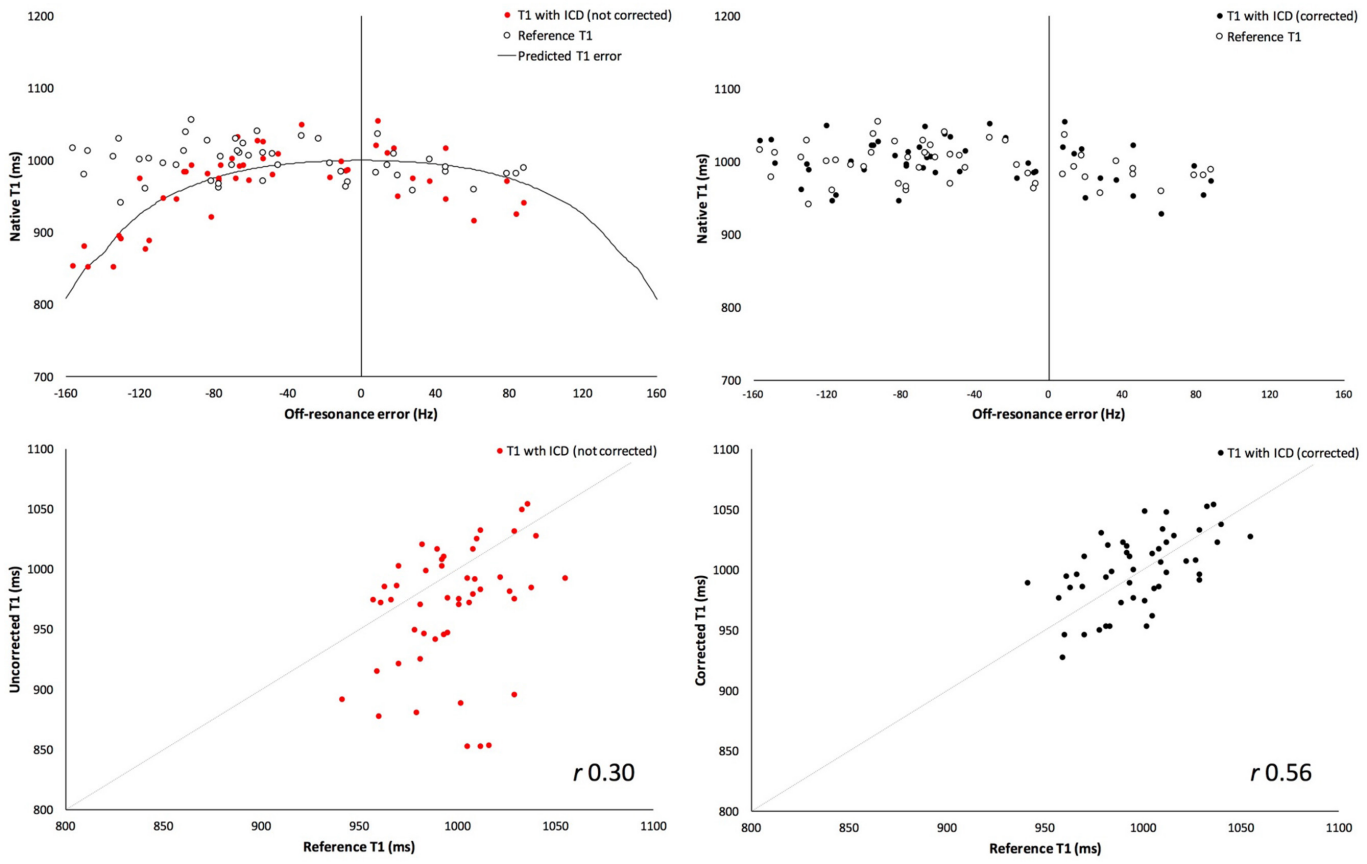
Validation of T1 measurement correction in healthy volunteers with externally-placed ICD generator. Uncorrected measured T1 values with externally-placed ICD generator (red) (**top**, left) shows greater underestimation of T1 values with increasing off-resonance frequency, compared to reference T1 (white). Reference T1 is the T1 measurement before generating metallic artifact. Following correction for off-resonance frequency (**top**, right), corrected T1 values (black) are similar to the reference T1 (white). **Bottom**: Correlation with true measured “reference” T1 values is significantly better when T1 values are corrected (right, black) than uncorrected (left, red) in the presence of an ICD.

Ten healthy volunteers were scanned, age 33 ± 2 years old and 80% male (BSA 1.9 ± 0.2 m^2^, heart rate 67 ± 9 beats per minute). An average of five out of six segments per volunteer were free of visual artifact. In the segments free of visual artifact, the mean segmental off-resonance frequency induced by the ICD was 70 ± 41 Hz, and the maximal segmental off-resonance frequency per volunteer was 113 ± 25 Hz. T1 in the presence of the ICD was underestimated compared to reference T1 by 30 ± 51 ms (967 ± 52 vs. 997 ± 26 ms, respectively, *p* = 0.0001), and the offset was greater with larger off-resonance frequency (Figure 2). After correction for expected off-resonance using previously described Bloch simulation (Supplementary Table 1), corrected T1 in the presence of an ICD was similar to reference T1 (without an ICD) with a mean difference of 2 ± 27 ms (1,001 ± 31 vs. 997 ± 26 ms, respectively, *p* = 0.57).

The same analysis and correction was also applied to only those segments with large off-resonance error (>85 Hz, as defined above), *n* = 17 (32%) segments. There was greater underestimation of T1 compared to reference T1 by 87 ± 50 ms (919 ± 53 ms vs. 1,006 ± 24, respectively, *p* < 0.0001). Correction remained successful, and corrected T1 was similar to reference T1 with a mean difference of −6 ± 29 ms (1,000 ± 32 vs. 1,006 ± 24 ms, respectively, *p* = 0.45).

### Measured T1 Error in Patients With CIEDs

Twenty-four patients with CIEDs were scanned, age 54 ± 19 years old, 54% male. Implanted CIEDs were 11 (46%) permanent pacemakers (PPMs), 8 (33%) ILRs and 5 (21%) ICDs; 20 (83%) were MRI-conditional.

One patient with an ICD had completely non-diagnostic T1 mapping due to extensive banding artifact, and overall patients with ICDs had 53 ± 27% of segments with banding artifact. Patients with PPMs had 42 ± 31% of segments with banding artifact. One patient with an ILR had banding artifact, affecting two out of six segments.

In the segments free of visual artifact, the maximal absolute off-resonance frequency per patient was 114 (48–147) Hz. The anterior wall and anterior-septum had the greatest proportion of segments with significant frequency >85 Hz (42 and 50%, respectively), whilst the inferior wall had the least proportion (12.5%). The inferior-septum, inferior-lateral and lateral walls had significant frequency in 29, 20, and 33% of segments, respectively. T1 was underestimated in the presence of off-resonance frequency, and as predicted by Bloch simulation the T1 offset was greater with larger frequency, Supplementary Figure 2. The presence of a visible, in-slice RV lead cross-section correlated with greater off-resonance in the adjacent myocardial segment (*r* = 0.44, *p* = 0.03).

### Magnitude of T1 Error Correction Required in Patients With CIEDs

**Figure 3:**
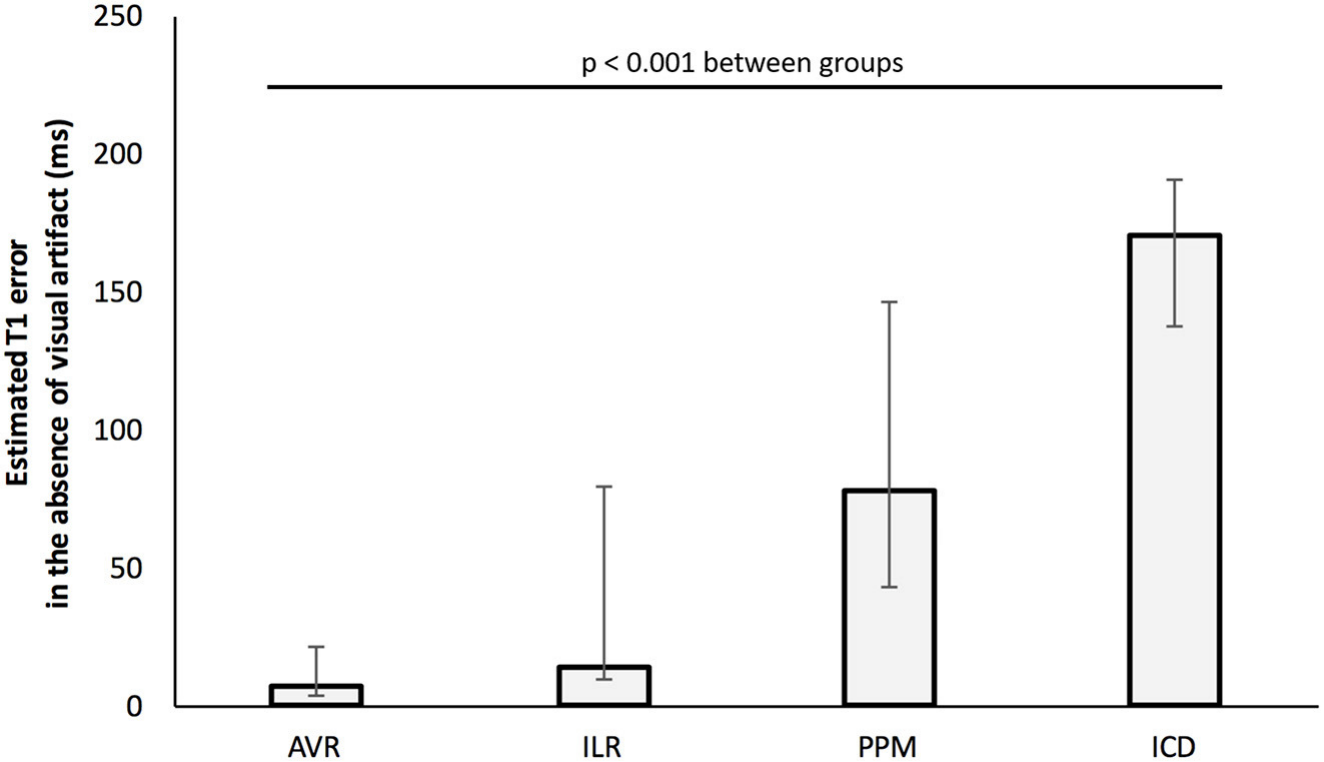
Maximal T1 error per patient by cardiac device (after exclusion of segments with visual artifact). Bars represent the median of the peak segmental off-resonance induced T1 error for individual patients. Confidence bars represent group maximum and minimum peak errors.

**Figure 4:**
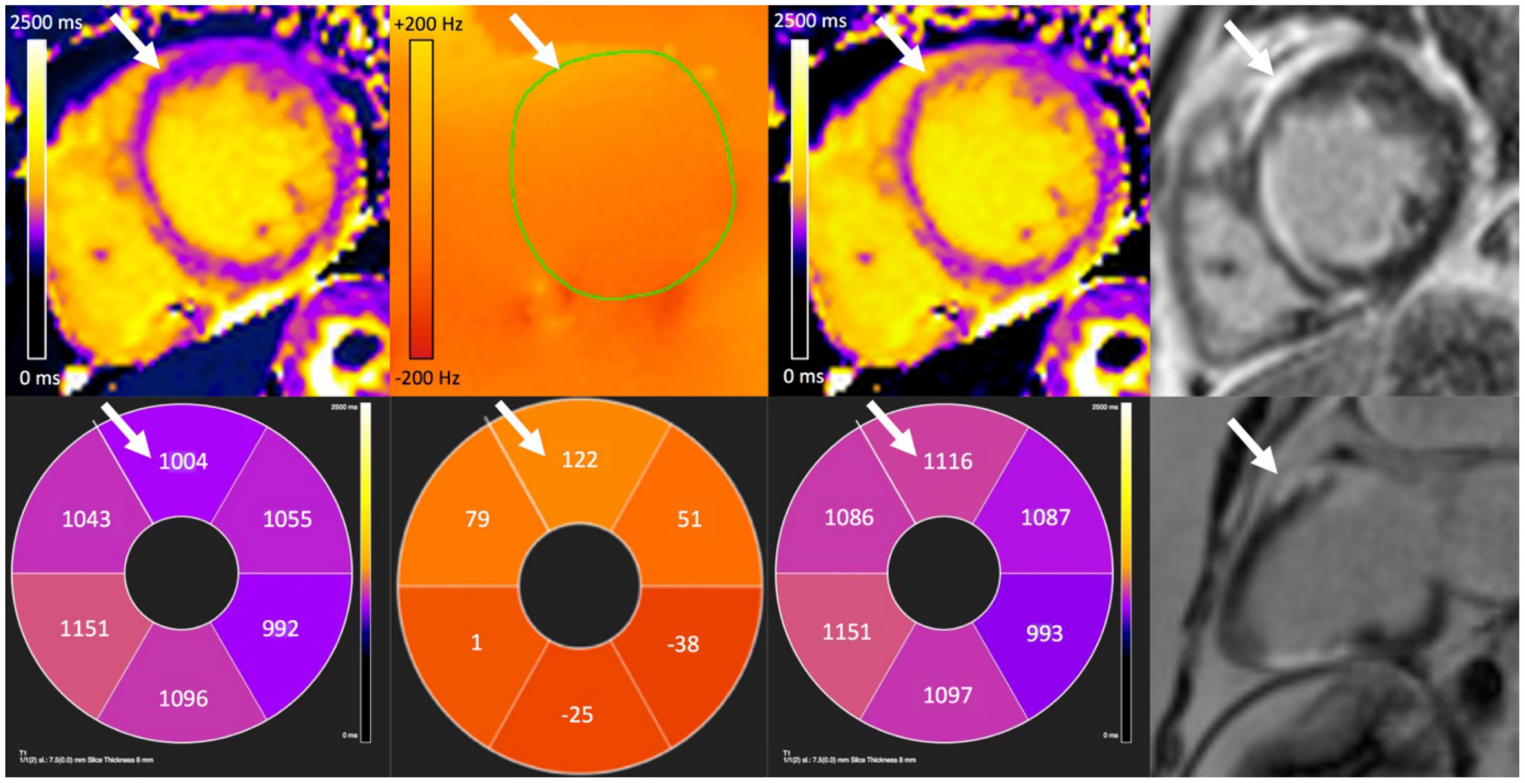
A corrected T1 map revealing anterior wall scar (white arrows) in a patient with cardiac sarcoidosis and an implanted permanent pacemaker. There is false lowering of original T1 measurement (far left) in the anterior wall due to off-resonance (center-left). Off-resonance can be measured and then corrected for to generate an off-line T1 map (center-right). Bottom row shows corresponding average segmental T1 and off-resonance (Hz) values, and 2-chamber wideband late gadolinium enhancement (LGE). Elevated T1 after correction corresponds with distribution of scar seen on wideband LGE (far right) imaging.

**Figure 5:**
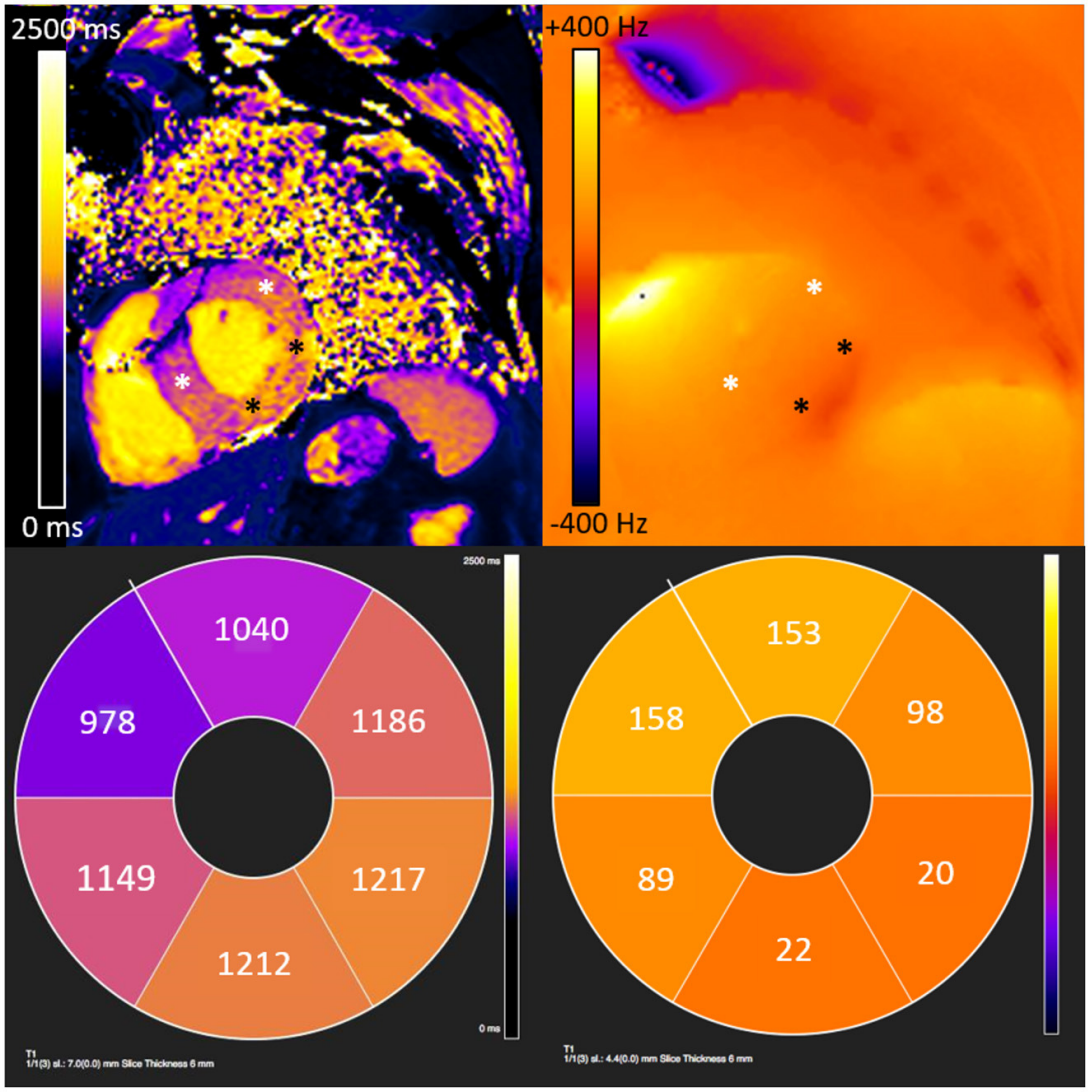
Underestimation of measured T1 values due to off-resonance induced error extends beyond visually apparent artifact in a patient with cardiac amyloidosis and an implantable loop recorder. There is visible banding artifact in the anterior and antero-septal segments from an implantable loop recorder. However, extending beyond the visible artifact, there is significant off-resonance which causes underestimation of T1 values in the anterior, anterolateral and anteroseptal walls (white asterisk). The inferior and infero-lateral wall show limited off-resonance where T1 values are consistent with cardiac amyloidosis (black asterisk).

Correction of maximal off-resonance resulted in an increase in T1 per patient of 63 (7–143) ms. The greatest correction in T1 required for patients with ILRs, PPMs and ICDs was 79, 146, and 191 ms, respectively (Figure 3). Figure 4 demonstrates the changes visually and quantitatively in one patient with cardiac sarcoidosis and regional scar before and after voxel-wise correction for off-resonance is applied to the original T1 map. T1 correction was clinically useful, for example, one patient with suspected cardiac amyloidosis and an ILR, T1 was apparently high-normal due to off-resonance, however, after correction, T1 was significantly elevated to >1,200 ms (Figure 5).

### Measured T1 Error in Patients With AVRs

Thirty-one patients with AVRs were scanned, age 69 ± 8 years. AVRs were 14 (45%) metallic; 17 (55%) bioprosthetic. No banding artifact was noted on T1 mapping.

The maximal absolute off-resonance frequency per patient was 48 ± 16 Hz. There were no segments with significant frequency >85 Hz. There was no difference between mechanical or bioprosthetic AVRs (*p* = 0.48). There was no gradient between basal, mid and apical short-axis orientation off-resonance (38 ± 16 Hz vs. 42 ± 17 Hz vs. 42 ± 15 Hz, *p* = 0.5).

## Discussion

This study demonstrates that there is significant error in measured myocardial T1 values in patients with cardiac implantable electronic devices, even when there is no visually detectable artifact. This is attributable to increased off-resonance frequency which can be detected on a single breath-hold acquisition and used to correct measured T1. T1 mapping is becoming a standard component of cardiovascular magnetic resonance imaging, particularly for patients with suspected cardiomyopathy or myocarditis. Many such patients have CIEDs and failure to detect T1 error can lead to mis-diagnosis of regional pathology or infiltrative cardiomyopathy.

Off-resonance reduces measured T1, with a peak error of 63 ms per patient with a CIED, a magnitude that is clinically important. Even after excluding visual artifact, T1 measurements were still significantly underestimated in half of patients with CIEDs in the anterior-septum, and approximately one-third of patients in the inferior and infero-lateral walls. Error was present even in some patients with smaller ILRs, and so off-resonance frequency should be considered in all patients with CIEDs undergoing T1 mapping. As expected, both the presence of larger CIEDs, and the proximity of an RV lead increased error (11). Error may also be relative to the distance of the CIED from the heart. Significant T1 measurement error was not detected in patients with aortic valve replacements and sternal wires, which may be attributable to the smaller amount of ferro-magnetic material within the implant. The quantification of off-resonance to detect T1 error may also be useful for other implants such as endovascular aortic repairs or atrial septal closure devices.

Application of an off-resonance frequency correction was feasible in patients with CIEDs, and accurate when validated in healthy volunteers with artifact generated by external placement of a CIED on the chest wall. Approximately half of AHA segments in patients with ICDs and PPMs demonstrated visual artifact, precluding further analysis. However, correction was possible for remaining myocardial segments in all but one patient (with an ICD). Accurate measurement in one or two segments should be sufficient to investigate global infiltrative pathologies such as cardiac amyloidosis and cardiac iron loading (where T2 star imaging is likely to be non-diagnostic due to visual artifact). Failure to correct for off-resonance risks underestimation of measured T1 values which, in the case of cardiac amyloidosis may risk misdiagnosis, or for patients with normal T1 over-diagnosis of Anderson-Fabry disease or iron loading.

Alternative strategies have been applied for T1 mapping in patients with CIEDs including adapting the sequence to include a wideband inversion pulse (21, 22). The benefits of wideband imaging appear to be greater in ICDs than ILRs or PPMs because of more prominent signal void and SSFP banding artifact (8), however the latter group forms the majority of CIEDs implanted worldwide. Additionally, MOLLI with spoiled gradient echo (GRE) readout is less prone to susceptibility artifact seen in SSFP imaging under off-resonance conditions and therefore may be more accurate than standard MOLLI sequences using SSFP readout (as used in this study) (23). However, these alternative sequences are less widely available than SSFP MOLLI, have lower signal-to-noise and larger voxel size and require additional reference ranges to be developed.

There are alternative approaches to decrease sensitivity to off-resonance in patients with CIEDs, at a cost of reduced spatial resolution and signal to noise. These include using a shorter echo time, lower flip angle or smaller matrix size (11). Application of voxel-wise T1 correction of off-resonance frequency as a separate tool may further improve diagnostic accuracy. This is described in principle here and accounts for different breath-hold depth and provides an intuitive visual map, but may be less scalable to other centers than a schema based on segmental measurement.

**Figure 6:**
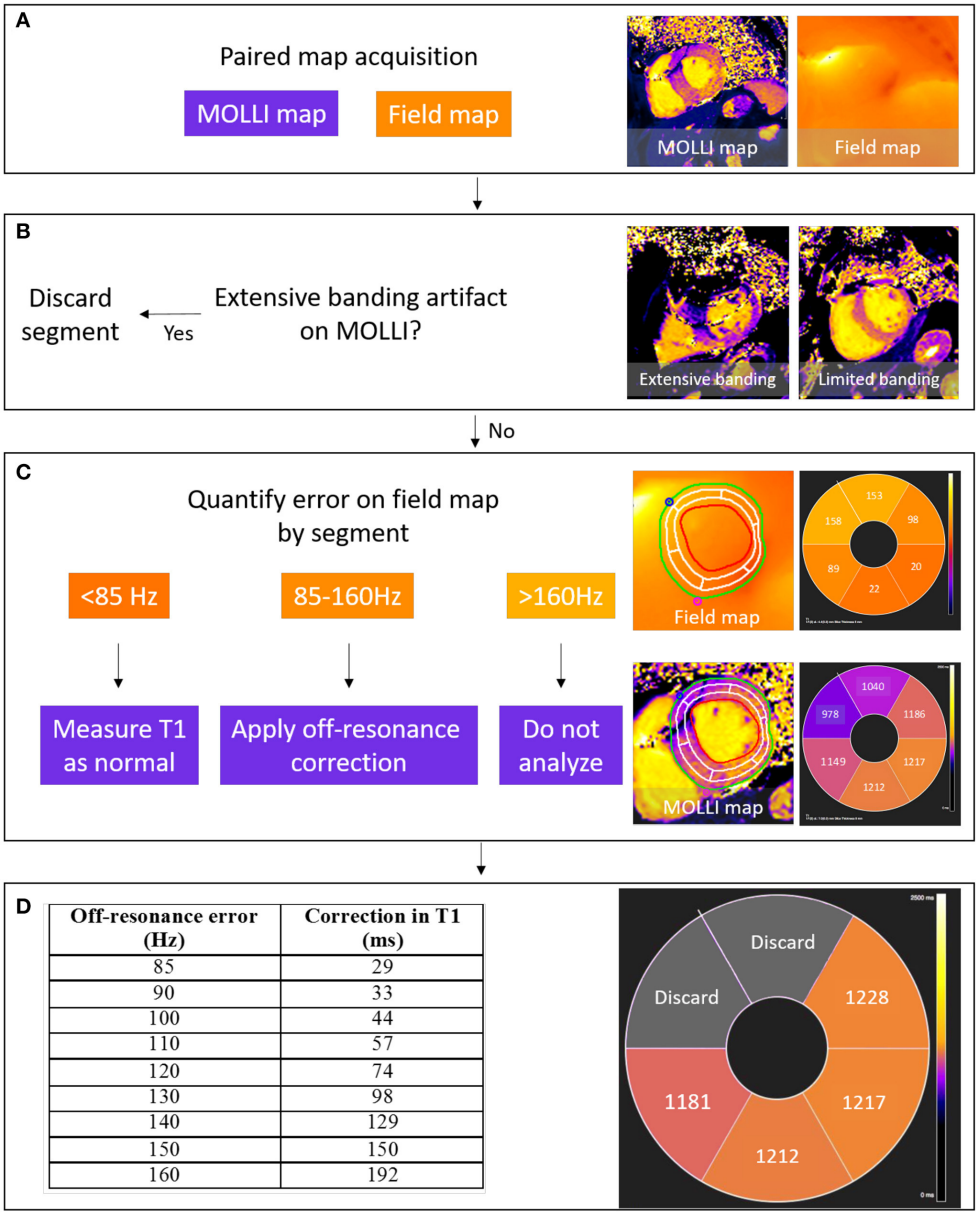
Schema for the detection and correction of off-resonance induced error in T1 mapping. A MOLLI T1 map is acquired, paired with a field map with the same field of view and slice position **(A)**. Segments with visible banding artifact are excluded from analysis **(B)** and then the field map is analyzed to quantify segmental off-resonance frequency in segments without artifact. Segmental T1 values are then measured from the MOLLI map and paired with segmental field map measurements of off-resonance using the right ventricular insertion points as fiducial markers for registration. If off-resonance frequency is low, T1 can be measured directly; if moderate error (85–160 Hz), Bloch simulated correction values assume a true T1 of 1,000 ms for a Siemens-specific 5(3s)3 MOLLI sampling scheme; and if high error, the segment is discarded **(C,D)**. Acceptable T1 error is defined by the inter-study standard deviation in T1 (28 ms), equivalent to an off-resonance <85 Hz.

Look-up curves for T1 correction may have wider applications to correct off-resonance attributable to other sources including hepatic iron, and tissue interfaces (14). By measuring T1 with and without CIED-induced artifact, these data demonstrated that *in-vivo* Bloch simulations accurately described the measured T1 underestimation. The correction curves improve measurement accuracy whilst maintaining similar precision. When comparing corrected T1 values to a reference T1 of the same patient, the sd of the residual difference was 27 ms, and is comparable to an inter-study difference sd of 28 ms (20). This cut-off for acceptable accuracy is equivalent to an off-resonance frequency of 85 Hz, and is incorporated into a proposed schema for conventional T1 MOLLI analysis in patients with CIEDs (Figure 6).

## Study Limitations

This technique cannot overcome SSFP banding artifacts, but can detect error in T1 estimation in the presence of metallic cardiovascular implants in AHA segments without banding artifact. It may also be incrementally useful in combination with T1 sequences using wideband inversion pulses. If different sample schemes are used or T1 values differ significantly from 1,000 ms, a modified correction curve can be used to maintain the highest accuracy (11). The schema proposed is to correct for segmental off-resonance T1 frequency, which demonstrated sufficient accuracy in a small cohort. Accuracy may be subject to movement error or respiratory variation. A voxel-wise field map correction as described should minimize these sources of inaccuracy. Errors in T1 measurement may also influence extra-cellular volume (ECV) calculation. As previously reported, the measurement of blood T1 is less sensitive to off-resonance error due to flow. The dominant source of error is myocardial T1, but previous analysis reported that bias in ECV measurement is relatively small, ~1% (in ECV percentage units) (11).

## Conclusions

T1 measurement in patients with cardiac implantable electronic devices using a conventional MOLLI sequence is unreliable even when there is no visual artifact. This is due to off-resonance frequency which can be quantified from single breath-hold field maps, and then used to correct T1 values accurately. This can help in the diagnosis of suspected infiltrative or inflammatory myocardial diseases in patients with CIEDs.

## Data Availability Statement

The original contributions presented in the study are included in the article/Supplementary Material, further inquiries can be directed to the corresponding author/s.

## Ethics Statement

The studies involving human participants were reviewed and approved by UK National Research Ethics Committee (07/H0715/101). The patients/participants provided their written informed consent to participate in this study.

## Author Contributions

AB drafted the manuscript and all co-authors critically reviewed and amended the manuscript. All authors contributed to this study by conceiving and designing the study, performing the data collection and the statistical analysis, or assisting in data interpretation.

## Conflict of Interest

The authors declare that the research was conducted in the absence of any commercial or financial relationships that could be construed as a potential conflict of interest. The handling Editor declared a past co-authorship with several of the authors JM and PK.
