# Supplementary material for: Measurement of T1 Mapping in Patients With Cardiac Devices: Off-Resonance Error Extends Beyond Visual Artifact but Can Be Quantified and Corrected
Source: Front Cardiovasc Med. 2021 Jan 29;8:631366. doi: 10.3389/fcvm.2021.631366 (PMC7878555; doi:10.3389/fcvm.2021.631366)
Supplement: Supplementary file 1 [file Data_Sheet_1.docx]

Supplementary Material

| **Off-resonance error (Hz)** | **Correction in T1 (ms)** |
| --- | --- |
| 0 | 0 |
| 20 | 1 |
| 40 | 5 |
| 60 | 12 |
| 80 | 25 |
| 100 | 44 |
| 120 | 74 |
| 140 | 129 |
| 160 | 192 |

Supplementary Table 1 Error in T1 for given off-resonance errors estimated from Bloch simulation. This represents samples from the complete correction curve which is estimated for a true myocardial T1 of 1000ms using a MOLLI 5s(3s)3s sample scheme.^1^


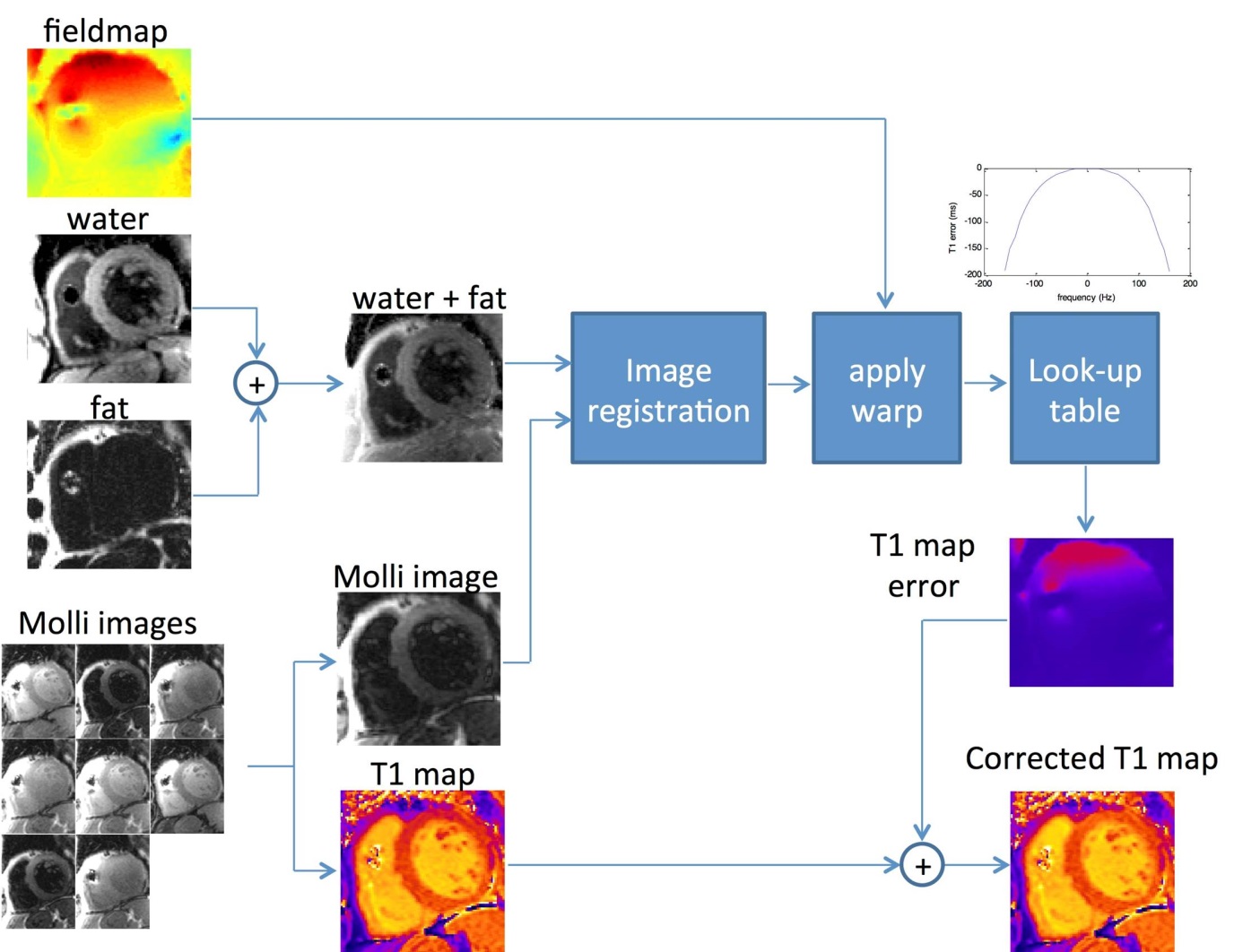


Supplementary Figure 1 Schematic for generation of T1 maps corrected for off-resonance error after co-registration using fat water and MOLLI imaging. Note the device lead in the RV, the off resonance at the top of the image on the field map and the increased T1 in the anterior segment of the corrected image.


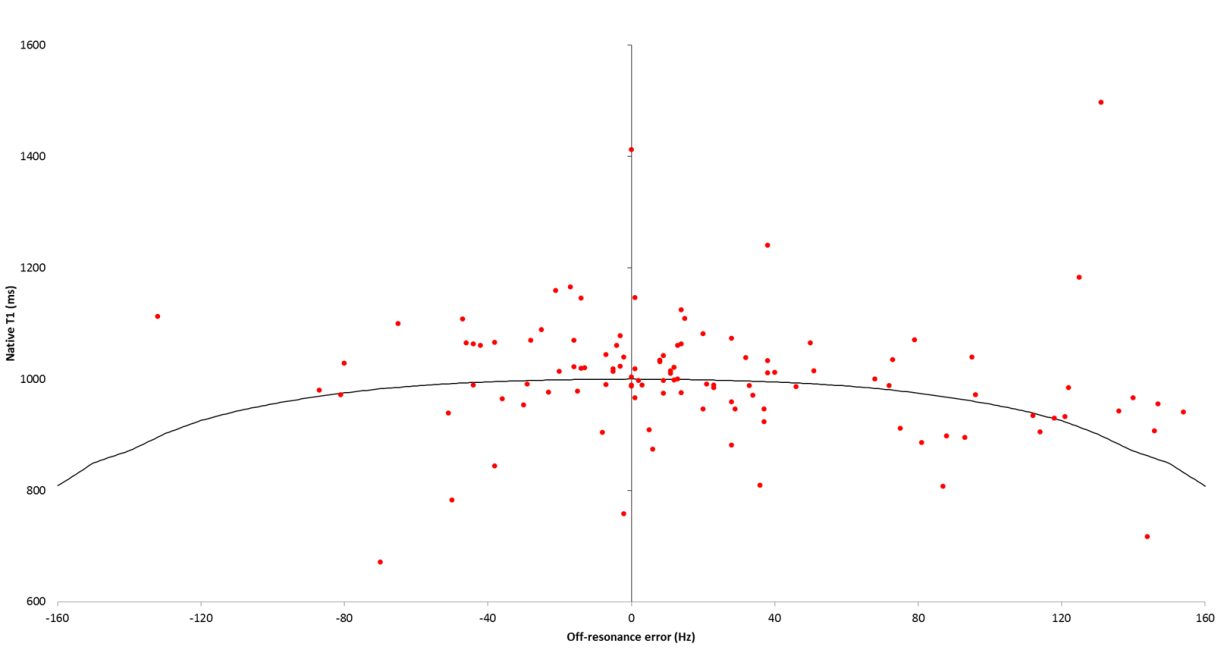


Supplementary Figure 2 Off-resonance error in patients with CIEDs is similar to Bloch simulation data (black line). The estimated off-resonance effect is for a true T1 of 1000ms, but as some patients may have higher or lower true T1, the apparent T1 may be appropriately lower or higher than predicted. Here, the outlier points are in patients with cardiac amyloidosis (high T1) or hemochromatosis (low T1).

**Supplementary References**

1. Kellman P, Herzka D, Arai AE, Hansen M. Influence of Off-resonance in myocardial T1-mapping using SSFP based MOLLI method. J Cardiovasc Magn Reson. 2013;15(1):63.
